# Supplementary material for: The Evolution and Characterization of the RNA Interference Pathways in Lophotrochozoa
Source: Genome Biol Evol. 2024 May 7;16(5):evae098. doi: 10.1093/gbe/evae098 (PMC11114477; doi:10.1093/gbe/evae098)
Supplement: evae098_Supplementary_Data [file evae098_supplementary_data.zip › Supplementary_materials.zip!/selected_miRNAs/NoGe_selected_miRNAs/known_miRNA_families_pdf_files/NMRB01000451.1_Notospermus_geniculatus_strain_Ushimado_scaffold451,_whole_genome_shotgun_sequence_13662.pdf]

Provisional ID : NMRB01000451.1_Notospermus_geniculatus_strain_Ushimado_scaffold451,_whole_genome_shotgun_sequence_13662
Score total : 133114.1
Score for star read(s) : 3.9
Score for read counts : 133105.8
Score for mfe : 1.4
Score for randfold : 0
Score for cons. seed : 3
Total read count : 261093
Mature read count : 260959
Loop read count : 0
Star read count : 134

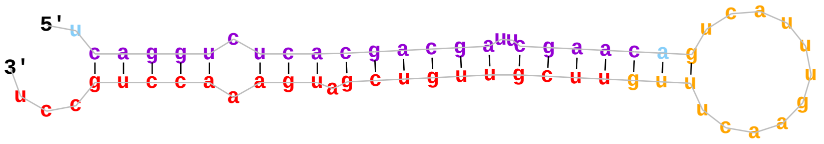

Genome coordinates: NMRB01000451.1 326750-326811

pre-miRNA sequence:
CAGGUCUCACGACGAUUCGAACCAGGUCUCACGACGAUUCGAACAGUCAUUUGAACUUUGUUCGUUGUCGAUGAAACCUGCCU

Mature sequence: UUCGUUGUCGAUGAAACCUGCCU

Aligned mature sequence of the *N. geniculatus* pre-miRNA with the top 5 hits from the BLAST search against the pre-miRNA mirGeneDB set:

```
                         ***********************
Nge_Mir-76               uucguugucgaugaaaccugccu
Npo-Mir-76_3p            uucguugucgaugaaaccugccu
```
